# The morphokinetic signature of human blastocysts with mosaicism and the clinical outcomes following transfer of embryos with low-level mosaicism

**DOI:** 10.1186/s13048-023-01324-w

**Published:** 2024-01-09

**Authors:** Yaoyu Zou, Yilun Sui, Jing Fu, Naidong Ge, Xiaoxi Sun, Yijuan Sun

**Affiliations:** Shanghai Ji Ai Genetics & IVF Institute, Obstetrics & Gynecology Hospital, Fudan University, Dalin Road, Shanghai, 200011 China

**Keywords:** Time-lapse, Mosaic embryo, Morphokinetics, Clinical outcome, Mosaicism type

## Abstract

**Background:**

Genetic mosaicism is commonly observed in human blastocysts. Embryos’ morphokinetic feature observed from time-lapse monitoring (TLM) is helpful to predict the embryos’ ploidy status in a non-invasive way. However, morphokinetic research on mosaic embryos is extremely limited. Moreover, transfer of mosaic embryos is a new attempt in reproductive medicine, while studies regarding the clinical and neonatal outcomes following transfer of embryos with different levels and types of mosaicism are needed. This study aimed to investigate the morphokinetic characteristics of mosaic blastocysts, uncover clinical outcomes of mosaic embryos, and evaluate the effect of level and type of mosaicism on transfer outcomes.

**Results:**

A total of 923 blastocysts from 229 preimplantation genetic testing cycles were cultured in TLM incubators in a single fertilization center between July 2016 and July 2021. Multivariate logistic regression models showed mosaic embryos had significantly shorter time to reach morula when compared with euploid (*P* = 0.002), mosaic with aneuploid (*P* = 0.005), and aneuploid (*P* = 0.005) embryos after adjusting the potential confounders. KIDScore is an artificial intelligence scoring program from time lapse incubation system to predict embryo implantation potential. Mosaic with aneuploid embryos had significantly lower KIDScore than euploid (*P* = 6.47e^−4^), mosaic (*P* = 0.005), and aneuploid (*P* = 0.004) embryos after adjustment. Meanwhile, we compared the clinical outcomes following transfer of low-level (< 50%) mosaic embryos (*N* = 60) with euploid embryos (*N* = 1301) matched using propensity scoring collected from September 2020 to January 2023. Mosaic embryos had significantly lower clinical pregnancy rate (41.67% vs. 57.65%, *P* = 0.015) and live birth rate (38.33% vs. 51.35%, *P* = 0.048) than the euploid embryos. Subgroup analyses showed the whole, segmental, and complex chromosome mosaic embryos had the similar clinical outcomes.

**Conclusions:**

The shortened time to reach morula in mosaic embryos and the low KIDScore in mosaic with aneuploid embryos revealed innovative clues to embryo selection with the non-invasive TLM and provided new insights into biological mechanism of chromosomal abnormality. The analyses of overall and subgroups of mosaic embryo transfer outcomes helped to optimize embryo transfer scheme for in-vitro fertilization procedures. Multi-center prospective studies with large sample sizes are warranted to validate our results in the future.

**Supplementary Information:**

The online version contains supplementary material available at 10.1186/s13048-023-01324-w.

## Background

Embryonic chromosomal abnormalities are the major causes of implantation failure, pregnancy loss, and birth defects in vitro fertilization (IVF). Since the advent of preimplantation genetic testing (PGT) in the early 1990s, aneuploid embryos with an altered copy number of the 23 chromosomes can be detected and discarded from transfer. Mosaicism, which is characterized by the presence of two or more chromosomally different cell constitutions within a single embryo, is a unique form of chromosomal abnormality and has been increasingly identified and quantified. The reported prevalence of mosaic embryos has a wide range due to the different testing methods, the threshold of reporting mosaicism, embryo culture systems, the number of cells biopsied and the inclusion criteria of the study populations [1–5]. By using the more sensitive assays such as next-generation sequencing (NGS) in PGT, the prevalence of mosaicism is reported to be 11% to 31% in the blastocysts [2, 3, 5].

Recently, an increasing number of studies have proposed that the non-invasive time-lapse monitoring (TLM) could be a promising method for assessing precise developmental events, such as morphokinetics, dysmorphisms, and abnormal cleavages, which helps to reflect the embryos’ developmental potential and predict their ploidy status by algorithms [6–8]. However, most studies investigating the correlation between embryonic morphokinetics and ploidy status did not independently analyze the data of mosaic embryos [7, 9, 10] probably due to the insufficiency for mosaicism detection of the platforms used in PGT; thus, mosaic embryos appear to be a vague area in the kinetic analysis. Considering a certain proportion of embryos are detected as mosaic by NGS, which exhibited distinct biological characteristics and clinical value compared with the euploid or aneuploid embryos, they should not be neglected in morphokinetic analysis. Moreover, the mosaicism might be a confounder which explains the inconsistency of kinetic markers identified in existing studies and the poor prognostic value of the ploidy predictive models built by different IVF centers [9–11]. As far as we know, there are only two studies reporting the morphokinetics of embryos with genetic mosaicism, but the results are inconsistent [12, 13]. Therefore, investigation of mosaic embryonic morphokinetics may help in drawing a consistent conclusion in embryo prediction and selection using TLM.

Since the first report of the healthy infants born following the transfer of mosaic embryos in 2015 [14], transferring mosaic embryos in IVF centers has become an option when no euploid embryos are available. However, existing studies regarding mosaic embryo transfer are limited in the sample size or lack the neonatal outcome follow-ups [15–20]. Moreover, whether the level [18] or type (mosaicism involving segmental, whole, or complex chromosomes) [21] of the mosaic embryos affects the clinical and neonatal outcomes is still conflicting [16–19]. The Preimplantation Genetic Diagnosis International Society (PGDIS) have suggested that transfer the mosaic blastocyst after appropriate consultation is one of the options for the patients without available euploid embryos, while transferring a mosaic embryo is not without increased risk compared to the transfer of a euploid embryo [22]. The American Society for Reproductive Medicine similarly stated that outcomes reported after transfer of an embryo with mosaic results seem to be reassuring; however, current data are limited and should be interpreted with caution due to the unknown chance for the occurrence of an adverse prenatal or pediatric outcome [18]. Therefore, some IVF centers are reluctant to transfer the mosaic embryos due to the concerns about the developmental potential of the mosaic embryos and the safety of the offspring. Thus, more studies are needed to clarify the association of mosaic embryo transfer with clinical and neonatal outcomes and which specific type of mosaic embryos can be transferred to obtain a healthy live birth.

In this study, we investigated the morphokinetic characteristics of the mosaic embryos observed using TLM and the clinical and neonatal outcomes of the transferred low-level mosaic embryos. The transfer outcomes of mosaic embryos were further analyzed by different mosaic types and levels. Our study not only helps in clinical ploidy prediction with non-invasive TLM system, sheds light on the etiology of the mitosis-originated mosaicism, but also provides a valuable reference for mosaic embryo transfer when euploid embryos are unavailable.

## Results

### Baseline characteristics of the embryos with different ploidy status

A total of 923 blastocysts obtained from 229 PGT cycles were included and analyzed, among which 386 (41.82%) were euploid, 99 (10.72%) were mosaic, 67 (7.26%) were mosaic with aneuploid, and 371 (40.20%) were aneuploid as detected by NGS. The baseline characteristics of the embryos in the four groups were shown in Table 1. Distribution of the PGT indications were significantly different among the four groups (*P* < 0.05). The baseline characteristics of the mosaic embryos were similar to the euploid embryos. However, mosaic embryos showed significantly higher basal estradiol and significantly shorter duration of stimulation when compared with the mosaic with aneuploid embryos [basal estradiol, 46.00 (33.00, 60.00) pg/ml vs. 37.00 (29.00, 52.00) pg/ml, *P* = 0.025; duration of stimulation, 9.00 (8.00, 10.00) vs. 10.00 (9.00, 12.00), *P* = 0.012] and the aneuploid embryos [basal estradiol, 46.00 (33.00, 60.00) pg/ml vs. 38.00 (30.00, 53.00) pg/ml, *P* = 0.015; duration of stimulation, 9.00 (8.00, 10.00) vs. 10.00 (9.00, 11.00), *P* = 0.001]. The mosaic embryos from patients undergoing PGT-A cycles showed significantly younger maternal age than the aneuploid embryos [33.00 (30.25, 36.75) year vs. 36.00 (31.00, 39.00) year, *P* = 0.027], while the age was similar among the four embryo groups for the patients undergoing PGT-SR cycles. Moreover, the mosaic embryos were obtained from cycles using significantly lower dose of gonadotropin than the aneuploid embryos [24.00 (16.00, 32.00) ampoules vs. 29.00 (19.00, 40.00) ampoules, *P* = 0.008]. The other baseline characteristics did not differ significantly between the groups.

**Table 1:** Baseline demographic and clinical characteristics of the blastocysts classified by ploidy status

	**Euploid (*N* = 386)**	**Mosaic (*N* = 99)**	**Mosaic with aneuploid (*N* = 67)**	**Aneuploid (*N* = 371)**	***P* value**
**PGT indication**					**4.10E-10**
Patients undergoing PGT-A (n = 486)	238 (61.66%)	68 (68.69%)	30 (44.78%)	150 (40.43%)	
Patients undergoing PGT-SR (n = 437)	148 (38.34%)	31 (31.31%)	37 (55.22%)	221 (59.57%)	
**Female age (year)**					
Patients undergoing PGT-A	33.00 (30.00, 36.00)^a^	33.00 (30.25, 36.75)^a^	34.00 (30.00, 38.00)^ab^	36.00 (31.00, 39.00)^b^	**1.07E-04**
Patients undergoing PGT-SR	30.00 (28.00, 32.00)	30.00 (27.00, 32.00)	30.00 (27.00, 32.00)	30.00 (27.50, 32.00)	0.977
**Male age (year)**					
Patients undergoing PGT-A	34.00 (31.00, 38.00)^a^	34.50 (31.25, 38.00)^ab^	35.50 (32.00, 39.50)^ab^	36.50 (32.75, 41.00)^b^	**0.001**
Patients undergoing PGT-SR	31.00 (29.00, 34.00)	32.00 (29.00, 33.00)	31.00 (28.00, 34.50)	31.00 (29.00, 34.00)	0.847
**BMI (kg/m**^**2**^**)**	21.21 (19.53, 22.96)	21.48 (19.53, 22.94)	21.25 (19.71, 22.89)	22.03 (20.03, 23.31)	0.076
**Basal estradiol (pg/ml)**	40.50 (31.00, 55.00)^ab^	46.00 (33.00, 60.00)^a^	37.00 (29.00, 52.00)^b^	38.00 (30.00, 53.00)^b^	**0.008**
**Basal progesterone (ng/ml)**	0.50 (0.30, 0.80)	0.50 (0.40, 0.80)	0.55 (0.40, 0.80)	0.50 (0.30, 0.80)	0.964
**Basal luteinizing hormone (mIU/ml)**	4.50 (3.30, 6.40)	4.30 (3.40, 6.90)	4.30 (3.10, 5.40)	4.30 (3.40, 5.80)	0.260
**Basal follicle-stimulating hormone (mIU/ml)**	7.40 (6.20, 8.30)	7.40 (6.30, 8.10)	7.50 (6.30, 8.30)	7.30 (6.10, 8.30)	0.716
**Antral follicle count**	17.00 (13.00, 23.00)	16.00 (13.00, 24.00)	16.00 (13.00, 22.00)	19.00 (13.00, 23.00)	0.251
**Ovarian stimulation protocol**					0.303
Short GnRH agonist protocol	213 (55.18%)	48 (48.48%)	36 (53.73%)	218 (58.76%)	
GnRH antagonist protocol	173 (44.82%)	51 (51.52%)	31 (46.27%)	153 (41.24%)	
**No. of controlled ovarian stimulation cycles**	1.00 (1.00, 2.00)	1.00 (1.00, 2.00)	1.00 (1.00, 2.00)	1.00 (1.00, 2.00)	0.974
**Gonadotropin dose **(ampoules, 75 IU/ampoule)	26.50 (18.00, 33.00)^a^	24.00 (16.00, 32.00)^a^	30.00 (20.00, 40.00)^ab^	29.00 (19.00, 40.00)^b^	**0.001**
**During of stimulation days**	10.00 (9.00, 11.00)^ab^	9.00 (8.00, 10.00)^a^	10.00 (9.00, 12.00)^b^	10.00 (9.00, 11.00)^b^	**0.002**

Data are presented as Median (interquartile range) for continuous variable or Number (%) for categorical variable

*P* values are calculated using Kruskal–Wallis tests and Bonferroni post hoc for continuous variable and χ2 tests for categorical variable

Bolded *P* values reached statistical significance

Median with common superscripts across columns are not significantly different

*PGT-A* preimplantation genetic testing for aneuploidy, *PGT-SR* preimplantation genetic testing for structural rearrangements

### Morphokinetic parameters and dysmorphisms of embryos classified by different ploidy status

**Fig. 1:**
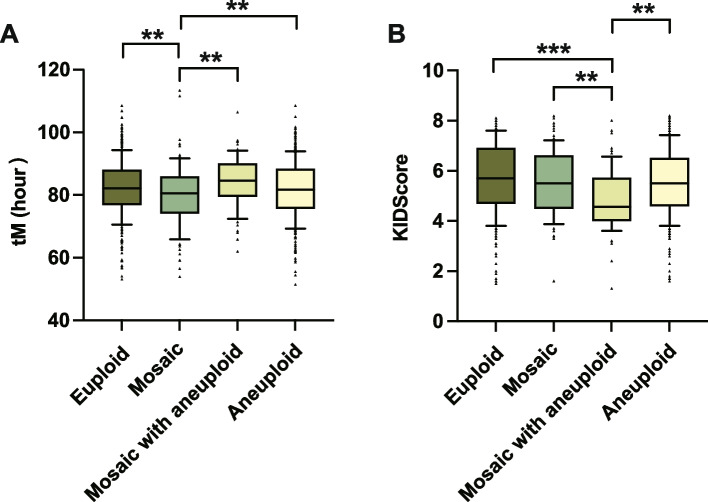
Distribution of morphokinetic parameter in euploid, mosaic, mosaic with aneuploid, and aneuploid embryos. The comparison of parameters tM (**A**) and KIDScore (**B**) were conducted using multivariate logistic regression with the adjustment of confounding factors. Boxplot shows the median with 10–90 percentiles. tM, time for morula. **, *P* < 0.001. ***, *P* < 0.001

Eighteen morphokinetic parameters based on TLM were analyzed among the embryos of the four different ploidy status (Table 2). The mosaic embryos exhibited significantly shorter tM than the embryos in the mosaic with aneuploid group [80.49 (74.24, 85.81) h vs. 84.57 (79.81, 89.95) h, *P* = 0.019]. Moreover, multivariate logistic regression models showed the mosaic group had significancy shorter tM when compared with the euploid (*P* = 0.002), mosaic with aneuploid (*P* = 0.005), and aneuploid (*P* = 0.005) embryos after adjusting the potential confounders (Table 3, Fig. 1A).

**Table 2:** Comparison of the morphokinetic parameters and the incidence of morphological dysmorphisms and irregular cleavages among embryos classified by ploidy status

	**Euploid**	**Mosaic**	**Mosaic with aneuploid**	**Aneuploid**	***P* value**
**tPB2 (h)**	3.37 (2.90, 4.00)	3.40 (3.30, 4.06)	3.13 (2.68, 4.23)	3.50 (3.91, 4.14)	0.275
**tPNa (h)**	9,73 (8.48, 11.48)	9.46 (8.21, 10.98)	9.72 (8.37, 11.67)	9.90 (8.50, 11.57)	0.204
**tPNf (h)**	22.38 (20.75, 23.89)	22.12 (20.72, 23.75)	22.67 (20.99, 24.47)	22.36 (20.68, 24.28)	0.339
**t2 (h)**	24.80 (23.12, 26.45)	24.37 (23.08, 26.14)	25.17 (23.89, 27.11)	24.80 (23.10, 26.73)	0.075
**t3 (h)**	35.54 (33.24, 37.77)	35.56 (33.53, 37.33)	35.93 (34.05, 38.09)	35.38 (33.14, 38.02)	0.564
**t4 (h)**	36.35 (34.41, 38.65)	36.41 (34.16, 38.53)	37.24 (35.19, 39.25)	36.54 (34.26, 39.10)	0.327
**t5 (h)**	49.27 (45.48, 52.59)	49.67 (45.91, 52.68)	49.45 (45.20, 53.04)	49.16 (45.13, 53.00)	0.888
**t6 (h)**	51.07 (47.45, 54.64)	51.77 (47.57, 55.01)	51.48 (48.10, 56.38)	50.82 (47.25, 54.97)	0.618
**t7 (h)**	53.17 (49.48, 57.36)	52.91 (48.58, 57.01)	53.96 (50.36, 59.83)	52.49 (48.47, 56.92)	0.119
**t8 (h)**	55.97 (51.17, 61.89)^ab^	56.72 (51.47, 62.89)^ab^	59.67 (52.54, 67.20)^a^	55.00 (50.69, 61.09)^b^	**0.040**
**tC (h)**	74.66 (66.28, 81.12)	73.33 (67.49, 809.55)	77.41 (69.17, 81.54)	73.16 (65.81, 80.23)	0.238
**tM (h)**	82.17 (76.89, 87.89)^ab^	80.49 (74.24, 85.81)^a^	84.57 (79.81, 89.95)^b^	81.77 (75.70, 88.29)^ab^	**0.027**
**tSB (h)**	93.31 (88.04, 100.02)	93.74 (87.89, 98.90)	97.95 (90.05, 101.93)	93.71 (88.30, 100.49)	0.135
**tHB (h)**	104.34 (97.96, 111.20)^a^	104.90 (99.77, 113.21)^ab^	110.14 (102.30, 113.51)^b^	105.44 (98.94, 111.05)^ab^	**0.030**
**S2 (h)**	0.50 (0.25, 1.00)	0.50 (0.25, 1.00)	0.50 (0.25, 1.25)	0.50 (0.25, 1.25)	0.775
**S3 (h)**	5.25 (3.00, 14.25)^ab^	6.00 (3.00, 11.26)^ab^	10.18 (4.34, 15.45)^a^	4.50 (2.75, 10.75)^b^	**0.005**
**CC3 (h)**	13.59 (12.09, 15.52)	14.02 (12.27, 15.78)	14.00 (12.25, 15.51)	13.50 (12.01, 15.25)	0.468
**KIDScore**	5.70 (4.70, 6.90)^b^	5.50 (4.50, 6.60)^b^	4.55 (4.00, 5.70)^a^	5.50 (4.60, 6.50)^b^	**3.60E-05**
**MN2**					0.289
Yes	112 (29.02%)	21 (21.21%)	23 (34.33%)	106 (28.57%)	
No	274 (70.98%)	78 (78.79%)	44 (65.67%)	265 (71.43%)	
**Frag-2**					0.177
Yes	59 (15.28%)	14 (14.14%)	6 (8.96%)	39 (10.51%)	
No	327 (84.72%)	85 (85.86%)	61 (91.04%)	332 (89.49%)	
**Direct cleavage**					0.500
Yes	19 (4.92%)	4 (4.04%)	6 (8.96%)	18 (4.85%)	
No	367 (95.08%)	95 (95.96%)	61 (91.04%)	353 (95.15%)	
**Uneven cleavage-2**					0.177
Yes	33 (8.55%)	7 (7.07%)	10 (14.93%)	26 (7.01%)	
No	353 (91.45%)	92 (92.93%)	57 (85.03%)	345 (92.99%)	
**Reverse cleavage**					**6.63E-06**
Yes	0 (0%)	3 (3.03%)	2 (2.99%)	12 (3.23%)	
No	386 (100.00%)	96 (96.97%)	65 (97.01%)	359 (96.77%)	

Data are presented as Median (interquartile range) for continuous variable or Number (%) for categorical variable

*P* values were calculated using Kruskal–Wallis tests and Bonferroni post hoc for continuous variable and χ2 tests for categorical variable

Median with common superscripts across columns are not significantly different

Bolded *P* values reached statistical significance

*tPB2* time of polar body emission, *tPNa* time of pronuclei appearance, *tPNf* time of pronuclear fade out, *t2*, *t3*, *t4*, *t5*, *t6*, *t7*, *t8* the time to reach 2, 3, 4, 5, 6, 7,8 cells, *tC* time to get compacted, *tM* time to reach morula, *tSB* time of starting blastulation, *tHB* time of the hatched blastocyst, *s2*, *s3* time of the second, third synchrony; *cc3* time of the third cell cycle, *MN2* multinucleation at the 2-cell stage, *Frag-2* fragmentation at the 2-cell stage

**Table 3:** Multivariate logistic regression analysis to evaluate the effects of time-lapse parameters on embryonic ploidy status

Variables	Mosaic vs. euploid (ref)		Mosaic vs. mosaic with aneuploid (ref)		Mosaic vs. aneuploid (ref)	
	OR [95% CI]	*P* value	OR [95% CI]	*P* value	OR [95% CI]	*P* value
PGT indication (PGT-A vs. PGT-SR)	0.65 (1.10, 0.39)	0.109	0.22 (0.09, 0.56)	1.23E-03	0.20 (0.11, 0.37)	2.31E-07
Female age (year)	0.99 (0.90, 1.11)	0.940	1.08 (0.92, 1.28)	0.354	0.95 (0.85, 1.07)	0.415
Male age (year)	0.99 (0.93, 1.08)	0.965	0.95 (0.85, 1.05)	0.304	0.99 (0.89, 1.09)	0.779
Basal estradiol (pg/ml)	1.01 (0.99, 1.02)	0.062	1.03 (1.01, 1.05)	0.005	1.02 (1.00, 1.03)	0.009
During of stimulation days	0.81 (0.67, 0.99)	0.042	0.95 (0.69, 1.32)	0.762	0.89 (0.71, 1.23)	0.336
Gonadotropin dose (ampoules, 75 IU/ampoule)	0.99 (0.97, 1.02)	0.485	0.97 (0.92, 1.02)	0.217	0.96 (0.94, 0.99)	0.017
t8 (h)	1.04 (0.99, 1.09)	0.112	1.02 (0.94, 1.10)	0.662	1.04 (0.99, 1.09)	0.063
tM (h)	0.95 (0.92, 0.98)	0.002	0.92 (0.87, 0.98)	0.005	0.95 (0.92, 0.99)	0.005
tHB (h)	1.03 (0.99, 1.07)	0.203	1.06 (0.99, 1.14)	0.086	1.02 (0.98, 1.07)	0.265
S3 (h)	0.99 (0.94, 1.04)	0.603	1.00 (0.93, 1.08)	0.945	1.01 (0.96, 1.06)	0.726
KIDScore	0.93 (0.74, 1.18)	0.551	1.88 (1.21, 2.91)	0.005	1.07 (0.84, 1.36)	0.582
Reverse cleavage (no vs. yes)	9.36E6 (1.71E6, 5.13E7)	< 0.001	3.20 (0.19, 54.96)	0.423	1.77 (0.21, 6.65)	0.854
Variables	Mosaic with aneuploid vs. euploid (ref)		Mosaic with aneuploid vs. aneuploid (ref)		Aneuploid vs. euploid (ref)	
	OR [95% CI]	*P* value	OR [95% CI]	*P* value	OR [95% CI]	*P* value
PGT indication (PGT-A vs. PGT-SR)	2.27 (1.22, 4.35)	0.009	1.33 (0.68, 2.62)	0.404	3.32 (2.32, 3.32)	4.48E-11
Female age (year)	0.98 (0.87, 1.10)	0.722	0.93 (0.83, 1.04)	0.930	1.07 (1.00, 1.14)	0.039
Male age (year)	1.03 (0.96, 1.11)	0.371	1.05 (0.97, 1.14)	0.209	0.98 (0.93, 1.03)	0.441
Basal estradiol (pg/ml)	0.99 (0.97, 1.00)	0.107	0.99 (0.98, 1.01)	0.405	0.99 (0.98, 1.00)	0.042
During of stimulation days	0.96 (0.77, 1.20)	0.714	1.03 (0.81, 1.31)	0.810	0.90 (0.79, 1.02)	0.101
Gonadotropin dose (ampoules)	1.01 (0.98, 1.05)	0.440	0.99 (0.96, 1.02)	0.411	1.03 (1.01, 1.05)	4.07E-04
t8 (h)	1.02 (0.97, 1.07)	0.534	1.01 (0.96, 1.06)	0.771	1.00 (0.97, 1.03)	0.99
tM (h)	0.99 (0.95, 1.03)	0.464	0.99 (0.96, 1.04)	0.889	0.99 (0.97, 1.02)	0.528
tHB (h)	1.00 (0.95, 1.04)	0.843	1.00 (0.96, 1.05)	0.897	1.00 (9.78, 1.03)	0.85
S3 (h)	1.00 (0.95, 1.05)	0.904	1.03 (0.98, 1.08)	0.215	0.97 (0.94, 0.99)	0.036
KIDScore	0.62 (0.48, 0.82)	6.47E-04	0.67 (0.51, 1.88)	0.004	0.92 (0.80, 1.07)	0.297
Reverse cleavage (no vs. yes)	7.46E6 (8.85E5, 6.33E7)	< 0.001	1.72 (0.20, 14.79)	0.622	-	-

*P* values were calculated using multivariate logistic regression analysis

Bolded *P* values reached statistical significance

*t2* the time to reach 8 cells, *tM* time to reach morula, *tHB* time of the hatched blastocyst, *s3* time of the third synchrony

The embryos in the mosaic with aneuploid group showed significantly delay in t8 [59.67 (52.54, 67.20) h vs. 55.00 (50.69, 61.09) h, *P* = 0.032] and s3 [10.18 (4.34, 15.45) h vs. 4.50 (2.75, 10.75) h, *P* = 0.006] than the aneuploid embryos, and significantly longer tHB [110.14 (102.30, 113.51) h vs. 104.34 (97.96, 111.20) h, *P* = 0.020] than the euploid embryos (Table 2), while the significance diminished with the covariates adjusted (*P* > 0.05, Table 3). The KIDScore was significantly lower for the mosaic with aneuploid embryos [4.55 (4.00, 5.70) h] than euploid [5.70 (4.70, 6.90) h], mosaic [5.50 (4.50, 6.60) h], and aneuploid [5.50 (4.60, 6.50) h] embryos (Table 2). Moreover, these differences remained significant when compared with euploid (*P* = 6.47e^−4^), mosaic (*P* = 0.005), and aneuploid (*P* = 0.004) embryos after adjusting for the potential confounders (Table 3, Fig. 1B).

Five types of dysmorphism and irregular cleavages were observed in this study (Table 2). The percentage of reverse cleavage was significantly lower in the euploid embryos (0, 0%) than in the mosaic (3, 3.03%, *P* = 0.049) and the aneuploid (12, 3.23%, *P* = 0.001) embryos. The MN2, Frag-2, direct cleavage and uneven cleavage-2 were not significantly different between the four groups.

### Clinical and neonatal outcomes of euploid and low-level mosaic embryo transfers

A total of 63 low-level mosaic embryos and 2137 euploid embryos were included in the study period. After propensity score matching (PSM), 60 mosaic embryos were matched to 1301 euploid embryos and the characteristics of the transfer cycles were shown in Table 4. The implantation rate was similar between the euploid and the mosaic embryos in the total age group, while this rate was significantly lower in mosaic embryos than euploid embryos among older women (33.33% vs. 62.46%,* P* = 0.024). The clinical pregnancy rate (57.65% vs 41.67%, *P* = 0.015) and live birth rate (51.35% vs 38.33%, *P* = 0.048) of the euploid group were significantly higher than those in mosaic group. Meanwhile, the euploid embryos had lower ectopic pregnancy rate than the mosaic embryos (0% vs. 4.00%, *P* = 0.032). No significant difference was found in the miscarriage rate, gestational age at delivery, birth weight, and congenital anomalies rate (Table 4).

**Table 4:** Clinical and live birth outcomes of single euploid and mosaic embryo transfers in young and advanced age groups

	**Total ages**		**< 38 years**		**≥ 38 years**	
	**Euploid embryos (*N* = 1301)**	**Mosaic embryos (*N* = 60)**	**Euploid embryos (*N* = 1008)**	**Mosaic embryos (*N* = 45)**	**Euploid embryos (*N* = 293)**	**Mosaic embryos (*N* = 15)**
**PGT indication**						
No. of patients undergoing PGT-A	805 (61.88%)	37 (61.67%)	546 (54.17%)	23 (51.11%)	259 (88.40%)	14 (93.33%)
No. of patients undergoing PGT-SR	496 (38.12%)	23 (38.33%)	462 (45.83%)	22 (48.89%)	34 (11.60%)	1 (6.67%)
**Antral ovarian follicle count**	15.00 (10.00, 21.00)	14.00 (9.00, 21.00)	15.00 (10.00, 21.00)	16.00 (9.00, 21.00)	12.00 (8.00, 18.00)	12.00 (7.50, 18.00)
**Female age at controlled ovarian stimulation (year)**	33.00 (30.00, 36.00)	33.00 (31.00, 36.25)	32.00 (29.00, 34.00)	32.00 (31.00, 34.00)	39.00 (38.00, 40.00)	40.00 (38.50, 40.00)
**Female age at transfer cycle (year)**	34.00 (31.00, 37.00)	34.00 (32.00, 37.25)	32.00 (30.00, 34.00)	32.00 (31.00, 35.00)	39.00 (39.00, 41.00)	40.00 (39.00, 41.50)
**No. of transfer cycles**	1.00 (1.00, 2.00)	2.00 (1.00, 2.00)	1.00 (1.00, 2.00)	2.00 (1.00, 2.00)	1.00 (1.00, 3.00)	2.00 (1.00, 2.00)
**Endometrial thickness at transfer (mm)**	8.00 (7.00, 10.00)	8.00 (7.00, 10.00)	9.00 (8.00, 10.00)	8.00 (8.00, 10.00)	8.00 (7.00, 9.00)	8.00 (6.50, 9.00)
**Implantation rate (%,no./total no.)**	68.49% (891/1301)	58.33% (35/60)	70.24% (708/1008)	66.67% (30/45)	62.46% (183/293)^a^	33.33% (5/15)^a^
**Clinical pregnancy (%,no./total no.)**	57.65% (750/1301)^b^	41.67% (25/60)^b^	59.92% (604/1008)	46.67% (21/45)	49.83% (146/293)	26.67% (4/15)
**Miscarriage rate (%,no./implantation no.)**	9.20% (82/891)	2.86% (1/35)	8.76% (62/708)	3.34% (1/30)	10.93% (20/183)	0% (0/5)
**Ectopic pregnancy rate (%,no./clinical pregnancy no.)**	0^c^	4.00% (1/25)^c^	0	4.76% (1/21)	0	0% (0/4)
**Live birth rate (%,no./total no.)**	51.35% (668/1301)^d^	38.33% (23/60)^d^	53.77% (542/1008)	42.22% (19/45)	43.00% (126/293)	26.67% (4/15)
**Gestational age at delivery (week)**	38.66 ± 1.82	38.83 ± 1.07	38.74 ± 1.85	38.88 ± 1.15	38.31 ± 1.68	38.81 ± 0.60
**Birth weight (g)**	3286.07 ± 520.90	3206.96 ± 443.01	3315.29 ± 526.61	3167.37 ± 471.53	3155.36 ± 532.67	3395.00 ± 219.32
**Congenital anomaly rate (%,no./total no.)**	0	4.35% (1/23)	0	5.26% (1/19)	0	0

Data are presented as Median (interquartile range) for continuous variable or proportion for categorical variable

*P* values were calculated using Mann–Whitney tests continuous variable and χ2 tests for categorical variable

a, b, c, d: *P* < 0.05

**Fig. 2:**
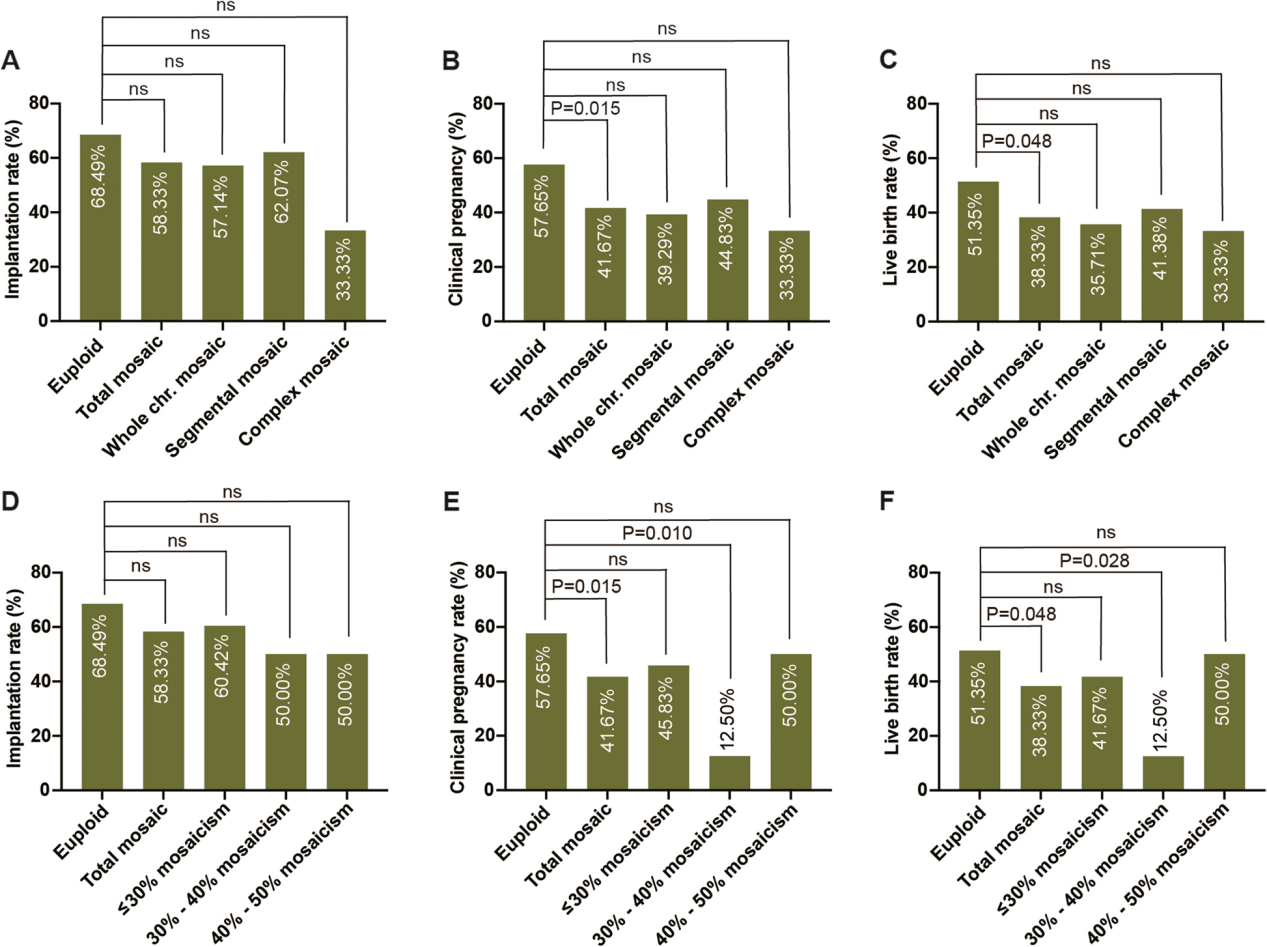
Effect of mosaicism type and level on clinical outcomes. Comparison of implantation rate (**A**), clinical pregnancy rate (**B**), and live birth rate (**C**) between euploid embryos with total, whole, segmental, and complex mosaic embryos. Comparison of implantation rate (**D**), clinical pregnancy rate (**E**), and live birth rate (**F**) between euploid embryos with total, ≤ 30%, 30%—40%, and 40%—50% mosaic embryos. The χ^2^ test was used at the 95% confidence level. Ns, no significance

No differences in the implantation rate, clinical pregnancy rate, or live birth rate were observed between the euploid embryos and any of the other subgroups of mosaic embryos (Fig. 2). Meanwhile, there was no significant difference among whole, segmental, and complex mosaic embryos. Besides, patients receiving the transfer of embryos with ≤ 30%, 30%—40%, and 40%—50% mosaicism had the similar clinical outcomes (Fig. 2).

## Discussion

Mosaicism is a common phenomenon observed in preimplantation embryos [4]. In this study, mosaic embryos were reported to have significantly shorter time to reach morula when compared with the euploid, mosaic with aneuploid, and aneuploid embryos. Meanwhile, mosaic with aneuploid embryos were found to have the significantly lower KIDScore than the euploid, mosaic, and aneuploid embryos. In addition, our study clearly demonstrated that low-level mosaic embryos can develop into live birth, which were not affected by the type or level of mosaicism, although their clinical pregnancy and live birth rate were significantly lower than euploid embryos. The study helped to improve the clinical application of the non-invasive TLM in embryo selection and provided more information of mosaic embryo transfer.

To the best of our knowledge, this is the first study to investigate the developmental morphokinetic signature of mosaic embryos in comparison with the euploid, mosaic, mosaic with aneuploid, and aneuploid embryos, and our studyreported a significantly decreased tM in the mosaic group than the other three groups. Although many studies have focused on the morphokinetic features of euploid and aneuploid embryos, embryos with mosaicism have always been neglected [7, 9, 10]. To date, only two studies have investigated the morphokinetic characteristics of mosaic embryos. The study by Martín et al. found that blastocysts with mosaicism had similar morphokinetics to both euploid embryos and aneuploid embryos [13]. Another research by Lee et al. reported that high-level (≥ 50%) mosaic blastocysts had prolonged t5, t8, and third cell cycle than the euploid embryos, while tM was similar between the euploid, low-level (< 50%) mosaic, high-level mosaic and aneuploid groups [12]. In the present study, we did not find a difference in morphokinetic timings between the high-level mosaic and euploid embryos as Lee et al. reported (data not shown), which might be due to the methodological differences, including the study population, the culture circumstances and hatching procedure of embryos, the time-lapse systems, and the mosaicism reporting criteria in PGT. However, our finding that the tM is significantly lower in mosaic embryos than the other three groups improves our knowledge of the biology of mosaicism. The morula stage is a crucial step for the acquisition and maintenance of reproductive competence during the development of preimplantation embryos [23] and tM is reported as an important predictor for pregnancy after embryo transfer [23–25]. Both delayed [26] and precocious compaction [27] during the morula stage have been reported to be detrimental for embryo developmental potential. As prolonged cell cycles in the human preimplantation embryo are likely to be associated with activated DNA repair processes, incorrect attachment of chromosomes to the spindle, or failure to complete previous phases of the cell cycle appropriately [28, 29], we consider that the unusually rapid tM may be related to inadequate cell cycle checkpoints in mosaic embryos and therefore leads to mitosis error and formation of mosaic embryos.

The KIDScore is a scoring program based on artificial intelligence (AI) for predicting embryo implantation after transfer, which was developed for time-lapse devices and derived from very large multicentric datasets based on morphokinetic timings including t8, tM, tHB, and s3 [30, 31]. Currently, only two studies have evaluated the Day 5 KIDScore predictive models, which reached similar conclusions. The study carried out in non-PGT embryos by Reignier et al. showed KIDScore were significantly associated with implantation rates after single blastocyst transfer [30]. A recent study by Gazzo et al. indicated that embryos with a higher KIDscore had an increased probability of being euploid and implanting [32]. Our study revealed that the KIDScore was significantly lower for the mosaic with aneuploid embryos than that of the other three groups. However, we did not find a significant difference in KIDScore between the mosaic, euploid and aneuploid embryos, which explains the predictive value of KIDScore models was moderate and perfectible in the study of Reignier et al. [30]. From a biological perspective, mosaic with aneuploid embryos contained significantly more abnormal chromosomes than aneuploid embryos (mean ± SD, 3.04 ± 1.62 vs. 1.69 ± 0.93, *P* = 1.02e^–7^), and theoretically extended the time for the self-correction process. We observed prolonged t8, tM, tHB, and s3 in mosaic with aneuploid embryos, while the significance diminished after adjustment of potential confounders. However, the KIDScore remained significantly lower in mosaic with aneuploid embryos than the other three groups after adjustment, implying the unique morphokinetic features of embryos with both meiotic and mitotic errors. Consequently, our results suggested that embryos of low KIDScore should be transferred with low priority during clinical embryo selection.

Studies regarding the clinical and neonatal outcomes after mosaic embryo transfer are still limited. Notably, the effect of the level and type of mosaicism on the transfer outcomes remains to be thoroughly evaluated. Overall, existing data revealed that mosaic blastocysts had the potential to develop into healthy live birth while exhibited significantly decreased possibility of ongoing pregnancies/live births and increased probability of miscarriage than the euploid embryos [15], and we reported the consistent results. The attenuated proliferation and preferential apoptosis of aneuploid cells along with the increased proliferation of euploid cells [33, 34] could be the reason why a live birth with normal chromosomes was obtained. Difference in implantation rate between euploid and mosaic embryos were not observed. However, mosaic embryos had a lower implantation rate than euploid embryos among women aged over 38 years, suggesting an impaired ability of self-correction for embryos from older women.

The evidence of the effect of mosaic level on the clinical outcomes are inconsistent. The studies by Spinella et al. [35] and Munné et al. [36] revealed that low-level mosaic embryos were associated with better clinical outcomes compared to high-level mosaic embryos. The PGDIS suggested that the higher-level mosaicism may be associated with less favorable outcomes compared with lower-level mosaicism [22]. However, Victor et al. [37] for the first-time reported transfers of blastocysts diagnosed as mosaic following NGS-based PGT-A, and their results did not demonstrate any correlation between the level of mosaicism and clinical outcome. In our study, only low-level (< 50%) mosaic embryos were transferred, and we did not find a difference between the mosaic level and transfer outcomes, which was similar to the research of Victor et al. [37] but inconsistent with the results of previous published studies [20, 35, 36]. We consider three possible reasons were: 1) as for low-level mosaic embryos, small difference of mosaicism level does not lead to significant changes in clinical outcomes; 2) the study of Victor et al. and ours adopted NGS-based PGT-A, which was more sensitive for mosaic detection; 3) the trophectoderm biopsy cannot always exactly reflect the rate of mosaicism for the entire blastocyst, especially when the difference of mosaicism level among embryos is relatively small.

The study of Munné et al. [38] indicated that embryos with single and double whole chromosomal mosaic aneuploidies as well as segmental mosaic structural aberrations showed comparable ongoing pregnancy rates. However, the study of Viotti et al. analyzed the transfer outcomes of 1,000 mosaic blastocysts and showed the type of mosaicism affected outcomes (segmental vs. complex mosaic, implantation: 51.6% vs. 30.4%; ongoing pregnancy: 43.1% vs. 20.8%) [20]. In our study, one out of three complex mosaic embryos succeeded to implant. And we found a similar tendency of more favorable clinical pregnancy and live birth outcomes in mosaic with segmental chromosomes involved, while the difference was not significant. We consider this was due to the limited sample size of transferred mosaic embryos. The PGDIS suggested that a decision to transfer a mosaic embryo can be prioritized either on the level of mosaicism or type of mosaicism [22]. However, studies with more participants are still warranted to explore embryo prioritization considering the level or type of mosaicism in the future.

In the study, we noticed one case of congenital anomaly in mosaic group. The fetus was found to have hemivertebra by ultrasonography and accepted an operation successfully at one year old. Our data showed that neonatal outcomes were similar between mosaic and euploid groups, which is consistent with studies of Zhang et al. and Lee et al. [17, 39]. Until now, no previous study has reported the newborn with congenital anomalies [17, 39]. The newborns have been usually healthy based on routine neonatal examination such as amniocentesis and prenatal ultrasonography, therefore congenital anomalies should not be a major concern in mosaic embryo transfer.

The study had some limitations. First, our findings based on a single-center might not be generalized to other IVF centers because of the heterogeneity among different IVF centers caused by the different patient demographics, embryo culture circumstances, biopsy protocols, genetic testing methods, et al. The sample size was also limited by the single-centre design of the study. Second, a single biopsy may not reflect the true mosaic status of the embryos due to the biology feature of mosaicism and may introduce variability and uncertainty to the findings, which was also a challenge to all studies regarding mosaic embryos and without a good solution so far. Therefore, it should be reminded that “the embryos with mosaicism” in the study are actually “the embryos diagnosed as mosaic embryos by current PGT technology”. However, this factor might be an explanation for the different development potential of the mosaic embryos, which highlighted the significance of our study exploring the non-invasive morphokinetic parameters with the developmental potential of the embryos diagnosed as mosaic with a single biopsy. Third, we did not link the specific morphokinetic characteristics of mosaic embryos (tM) to their transfer outcomes due to the retrospective design of the study, which was also a gap in existing studies. Therefore, future multicenter studies with a larger sample size are needed to validate our results and to evaluate the association of the morphokinetic features of mosaic embryos and their transfer outcomes.

## Conclusions

In conclusion, the study is the first to propose that mosaic embryos have the shortest time to reach morula, and mosaic with aneuploid embryos have the lowest KIDScore among the four groups. It is suggested that non-invasive TLM application has the potential to select mosaic embryos as well as exclude the untransferable mosaic with aneuploid embryos in clinical practice. Nowadays, some studies have shown the advantages of AI in processing TLM images/videos and assisting embryo selection, while the establishment of AI models are still based on the analysis of embryologists and the training of data set. Our study provides the evidence that there are differences in morphokinetic features among embryos with different ploidy status and meanwhile provides a better understanding of the biological mechanism for chromosomal abnormalities, and therefore helps to optimize the efficiency of the AI model for precise embryo selection in the future. Our observations with clinical outcome data and neonatal outcomes build on the concept that low-level mosaic embryos have the opportunity to achieve a healthy live birth. An optimal clinical management of mosaic embryos may be achieved after extensive genetic consulting. However, this single center-based study with a relatively small sample size might bring in the uncertainty of the results and influence the generalization to broader populations. In addition, selection bias could not be avoided in the retrospective part of the study. As a result, a multi-center prospective study with a large sample size is warranted to validate our results in the future.

## Methods

### Study design and population

This study consisted of two parts. Part one is a retrospective observational study to investigate the morphokinetic signature of mosaic embryos. Part two is a prospective case–control study to compare the transfer outcomes of mosaic embryos with euploid embryos.

For the first part of the study, we included all cycles with embryos cultured in the TLM system followed by NGS based PGT from July 2016 to July 2021 in Shanghai JiAi Genetics & IVF Institute, Obstetrics and Gynecology Hospital of Fudan University (TLM-NGS cohort). Each participant was required to have the normal 46,XX karyotype, representing the number of chromosomes was 46 and the sex chromosomes were both XX; and normal 46,XY karyotype for the male partner, meaning the number of chromosomes was 46 and the sex chromosomes were X and Y. The indications of PGT for aneuploidy (PGT-A, 53.65%) included advanced maternal age (≥ 38 years), repeated implantation failure, recurrent pregnancy loss, previous pregnancy loss due to aneuploidy, severe asthenospermia and oligospermia, and complex indications (more than one of the above indications). The indications for PGT for structural rearrangements (PGT-SR, 47.35%) included Robertsonian translocation, reciprocal translocation, inversion, chromosomal abnormalities, and mosaicism of the male or female partner. Embryos graded over 5BC or 5CB [40] at the blastocyst stage were biopsied for chromosomal analysis. To guarantee the accuracy of the collected TLM parameters, embryos that were not amenable to the TLM assessment because of excessive cytoplasmic fragmentation (> 50%) at the cleavage stage or poor video quality were excluded. For the second part of the study, all the low-level mosaic embryos (< 50%) and euploid embryos transferred in the same period served as the control group underwent PGT-A/PGT-SR from September 1st, 2020 to January 30th, 2023 were recruited to investigate the transfer outcomes. The exclusion criteria for the participants were as follows: 1) embryos underwent double vitrification or a second biopsy; 2) embryos underwent PGT for monogenic disorders; 3) biopsies were tested by other techniques, such as array comparative genomic hybridization or single-nucleotide polymorphism array.

Written informed consent was obtained from all the participants. The study was approved by the ethics committee of the Shanghai JiAi Genetics & IVF Institute (reference number: JIAI-E2017-10; approval date: May 8th, 2017).

### Embryological laboratory phase

Oocyte retrieval, denudation, and intracytoplasmic sperm injection (ICSI) were performed according to the routine clinical and laboratory procedures in our IVF institute [6]. Briefly, an antagonist protocol or GnRH agonist protocol for controlled ovarian hyperstimulation was used for each participant [6]. Oocyte retrieval was performed using transvaginal ultrasound-guided follicular aspiration, 36 h after human chorionic gonadotropin (Livzon Pharmaceutical Group, China) injection [41]. After the denudation of granulosa cells, ICSI were performed by experienced embryologists. Zygotes were individually incubated in a time-lapse incubator (EmbryoSlide, Vitrolife, Goteborg, Sweden) with G1 (D0-D3) and G2 (D3-D5/D6) (Vitrolife), 6% CO_2_, and 5% O_2_, at 37ºC, until day 5 or day 6. A small incision traversing the zona pellucida of each embryo was made on day 3 with laser (Hamilton-Thorne, USA). A trophectoderm (TE) biopsy and cryopreservation were performed at the blastocyst stage. Vitrification and warming of blastocysts were conducted using a Cryotop device and vitrification kit (KITAZATO, Shizuoka, Japan).

### TLM

A series of images were acquired in seven focal planes every 15 min, using a TLM machine (EmbryoScope, Vitrolife). The precise kinetic timings, embryonic dysmorphisms, and irregular cleavage events were annotated by two well-trained embryologists blinded to the ploidy status, using EmbryoViewer (Vitrolife). If the timing difference from the two embryologists was more than one hour, re-annotation would be done after discussion. The average of morphokinetic timings was used in this study. We defined the following precise timings and measured them post-mid-time of the ICSI microinjection operation, as previously described [6]. Morphokinetic parameters included the time of the second polar body emission (tPB2), time of two pronuclei (PN) appearance (tPNa), time of PN fading (tPNf), division time of two to eight blastomeres (t2 to t8), time of the first fusion of two blastomere membranes (tC), time when all cell boundaries were not obvious to form into morula (tM), time of starting blastulation (tSB), time of the hatched blastocyst (tHB), time of the third cell cycle (cc3 = t5–t3), time of the second synchrony (s2 = t4–t3), and time of the third synchrony (s3 = t8–t5). In addition, we observed the following dysmorphisms: multinucleation at the 2-cell stage (MN2), fragmentation at the 2-cell stage (> 25% cytoplasmic fragments, Frag-2), and uneven cleavage at the 2-cell/4-cell stage (> 25% uneven blastomere size, uneven-2/uneven-4). Single blastomeres dividing directly from one to three cells within 5 h (direct cleavage) and cleavage of reabsorbed blastomeres (reverse cleavage) was also observed. The KIDScore is an AI embryo scoring program using time lapse incubation system, and was initially written as “KIDScore, Vitrolife” and later as "KIDScore." This score can be obtained using the EmbryoViewer software, based on kinetic parameters, and has been proved to be associated with embryo implantation potential [30]. A kappa value that can test the inter-rater reliability of qualitative parameters was adopted for categorical variables. The kappa values for MN2, Frag-2, direct cleavage, uneven-2, uneven-4, and reverse cleavage were 0.97, 0.91, 0.95, 0.84, 0.88, 1.00, respectively.

### PGT testing and mosaicism classification

NGS is a platform that has been recognized as the most efficient in detecting mosaicism due to its superior resolution in both whole chromosomes and segmental regions [42]. The biopsies were analyzed for the ploidy status using NGS in accordance with our genetic laboratory guidelines [3]. Briefly, the whole genome DNA of TE biopsies was amplified and randomly fragmented, to construct a library using the pre-implantation genetic screening for aneuploidy kit (Berry Genomics Corp., Beijing, China). Purified libraries were pooled, denatured, and sequenced using a NextSeq CN500 sequencer (Illumina Inc.). All the sequencing reads were aligned to the human genome sequence (hg19). The size threshold for calling copy number variations (CNVs) was ≥ 3 Mb, and that for calling mosaic CNVs was ≥ 5 Mb.

The ploidy results of the samples were carefully analyzed and checked by two experienced technicians. Embryos that contained gain or loss of complete or segmental chromosomes in at least one of the 23 pairs of chromosomes were classified as aneuploid. Based on the diploid-aneuploid ratios detected by the NGS platform in the TE biopsy cells, blastocysts are classified as: (1) the “euploid embryo” group, representing the embryos with 0–20% of aneuploid cells, which means it includes euploid embryos without any chromosomal mosaicism and embryos with less than 20% mosaicism, as the 20% mosaicism is a detection threshold for NGS based PGT; (2) the “mosaic embryo” group, representing the embryos containing 20%–80% of aneuploid cells, which means it includes the embryos with low-level (20%–50%) mosaic embryos and high-level (50%–80%) mosaic embryos; (3) the “aneuploid embryo” group, representing embryo contains more than 80% of aneuploid cells. As the presence of two or more chromosomally different cell lines may also appear in aneuploid embryos, aneuploid embryos were further classified as mosaic with aneuploid embryos that implied a meiotic abnormality superimposed with post-zygotic mitotic abnormalities, and uniformly aneuploid embryos [3, 43]. In conclusion, the embryos for kinetic analysis were classified as euploid, mosaic, mosaic with aneuploid, and aneuploid embryos. In addition, mosaic embryos were subgrouped into whole (affecting exclusively whole chromosome abnormalities), segmental (affecting exclusively segmental chromosome abnormalities), and complex mosaic embryos (affecting more than one chromosome abnormalities) according to the involvement of chromosome structures and numbers for the evaluation of transfer outcomes.

### Embryo transfer and outcome parameters

In the clinical report, euploid embryos were recommended for transfer in priority, and low-level mosaic embryos containing > 20% and ≤ 50% abnormal cells were recommended to be retained and underwent genetic counseling before transfer. The priority of embryo transfer was determined according to chromosomal status firstly and the morphological grade secondly. A single blastocyst transfer was conducted when endometrial thickness reached 8 mm following hormone replacement treatment.

Embryo implantation was defined as positive serum β-HCG levels 14 days after embryo transfer. Clinical pregnancy was defined as visualization of the gestational sac on ultrasonography at 7 weeks of pregnancy. Ongoing pregnancy was confirmed when a pulsating fetal pole was present at 12 weeks of gestation. Miscarriage was defined as nonvisualization of the gestational sac at 7 weeks of pregnancy or pregnancy loss after 7 weeks of pregnancy. The rate of implantation, clinical pregnancy, and live birth was calculated per embryo transferred.

### Statistical analysis

Normal distribution of continuous variables was examined using histogram visualization and the Shapiro–Wilk test. As all parameters showed a skewed distribution, the variables were tested using non-parametric tests. Data are presented as median (interquartile range) for continuous variables or number (%) for categorical variables. The baseline characteristics of the embryos included were compared using the Wilcoxon rank–sum test. The Kruskal–Wallis test was adopted to compare the morphokinetic parameters among groups of embryos classified by ploidy status, followed by Bonferroni corrections. Categorical variables were compared using χ^2^ test or Fisher’s exact test. Logistic regression models were constructed to test the correlation between the baseline, morphokinetic parameters and ploidy status. Statistical significance was set at *P* < 0.05. The propensity score matching (PSM) was carried out using a caliper width of 0.2 of the standard deviation of the logit of the propensity score and 1:30 ratio by nearest neighbor matching, to better adjust for the potential confounding factors of cycle characteristics including PGT indication, antral follicle count, female age at controlled ovarian stimulation, female age at transfer cycle, number of transfer cycles, and endometrial thickness at transfer of the mosaic and euploid embryos. Analyses were carried out using SPSS version 26 (IBM Corporation) and figures were created using Prism version 9.3.1 (GraphPad Software).

### Supplementary Information

**Additional file 1:** **Supplementary table 4.** Clinical outcomes of the genotype of mosaicism after blastocyst transfers.

## Data Availability

The data used to support the findings of this study are available from the corresponding author upon request.

## Abbreviations

IVF: In vitro fertilization
PGT: Preimplantation genetic testing
NGS: Next-generation sequencing
PGT-A: PGT for aneuploidy
PGT-SR: PGT for structural rearrangements
TLM: Time-lapse monitoring
PGDIS: Preimplantation Genetic Diagnosis International Society
AI: Artificial intelligence
PSM: Propensity score matching
tPB2: Time of the second polar body emission
tPNa: Time of two pronuclei appearance
tPNf: Time of two pronuclei fading
t2, t3, t4, t5, t6, t7, t8: Division time of two to eight blastomeres
tC: Time of the first fusion of two blastomere membranes
tM: Time when all cell boundaries were not obvious to form into morula
tSB: Time of starting blastulation
tHB: Time of the hatched blastocyst
cc3: Time of the third cell cycle
s2, s3: Time of the second or third synchrony
MN2: Multinucleation at the 2-cell stage
Frag-2: Fragmentation at the 2-cell stage (> 25% cytoplasmic fragments)

## References

[1] Fragouli E, Alfarawati S, Daphnis DD, Goodall NN, Mania A, Griffiths T (2011). Cytogenetic analysis of human blastocysts with the use of FISH, CGH and aCGH: scientific data and technical evaluation. Hum Reprod.

[2] Nakhuda G, Jing C, Butler R, Guimond C, Hitkari J, Taylor E (2018). Frequencies of chromosome-specific mosaicisms in trophoectoderm biopsies detected by next-generation sequencing. Fertil Steril.

[3] Xiao M, Lei CX, Xi YP, Lu YL, Wu JP, Li XY (2021). Next-generation sequencing is more efficient at detecting mosaic embryos and improving pregnancy outcomes than single-nucleotide polymorphism array analysis. J Mol Diagn.

[4] Starostik MR, Sosina OA, McCoy RC (2020). Single-cell analysis of human embryos reveals diverse patterns of aneuploidy and mosaicism. Genome Res.

[5] Weissman A, Shoham G, Shoham Z, Fishel S, Leong M, Yaron Y (2017). Chromosomal mosaicism detected during preimplantation genetic screening: results of a worldwide Web-based survey. Fertil Steril.

[6] Zou Y, Pan Y, Ge N, Xu Y, Gu RH, Li ZC (2022). Can the combination of time-lapse parameters and clinical features predict embryonic ploidy status or implantation?. Reprod Biomed Online.

[7] Minasi MG, Colasante A, Riccio T, Ruberti A, Casciani V, Scarselli F (2016). Correlation between aneuploidy, standard morphology evaluation and morphokinetic development in 1730 biopsied blastocysts: a consecutive case series study. Hum Reprod.

[8] Viñals Gonzalez X, Odia R, Cawood S, Gaunt M, Saab W, Seshadri S (2018). Contraction behaviour reduces embryo competence in high-quality euploid blastocysts. J Assist Reprod Genet.

[9] Rienzi L, Capalbo A, Stoppa M, Romano S, Maggiulli R, Albricci L (2015). No evidence of association between blastocyst aneuploidy and morphokinetic assessment in a selected population of poor-prognosis patients: a longitudinal cohort study. Reprod Biomed Online.

[10] Zhang J, Tao W, Liu H, Yu G, Li M, Ma S (2017). Morphokinetic parameters from a time-lapse monitoring system cannot accurately predict the ploidy of embryos. J Assist Reprod Genet.

[11] Reignier A, Lammers J, Barriere P, Freour T (2018). Can time-lapse parameters predict embryo ploidy? A systematic review. Reprod Biomed Online.

[12] Lee CI, Chen CH, Huang CC, Cheng EH, Chen HH, Ho ST (2019). Embryo morphokinetics is potentially associated with clinical outcomes of single-embryo transfers in preimplantation genetic testing for aneuploidy cycles. Reprod Biomed Online.

[13] Martín Á, Rodrigo L, Beltrán D, Meseguer M, Rubio C, Mercader A (2021). The morphokinetic signature of mosaic embryos: evidence in support of their own genetic identity. Fertil Steril.

[14] Greco E, Minasi MG, Fiorentino F (2015). Healthy babies after intrauterine transfer of mosaic aneuploid blastocysts. N Engl J Med.

[15] Zhang YX, Chen JJ, Nabu S, Yeung QSY, Li Y, Tan JH (2020). The pregnancy outcome of mosaic embryo transfer: a prospective multicenter study and meta-analysis. Genes (Basel).

[16] Abhari S, Kawwass JF (2021). Pregnancy and neonatal outcomes after transfer of mosaic embryos: a review. J Clin Med.

[17] Lee CI, Cheng EH, Lee MS, Lin PY, Chen YC, Chen CH (2020). Healthy live births from transfer of low-mosaicism embryos after preimplantation genetic testing for aneuploidy. J Assist Reprod Genet.

[18] Practice Committee and Genetic Counseling Professional Group (GCPG) of the American Society for Reproductive Medicine (2020). Clinical management of mosaic results from preimplantation genetic testing for aneuploidy of blastocysts: a committee opinion. Fertil Steril.

[19] Lin PY, Lee CI, Cheng EH, Huang CC, Lee TH, Shih HH (2020). Clinical outcomes of single mosaic embryo transfer: high-level or low-level mosaic embryo, does it matter?. J Clin Med.

[20] Viotti M, Victor AR, Barnes FL, Zouves CG, Besser A, Grifo G (2021). Using outcome data from one thousand mosaic embryo transfers to formulate an embryo ranking system for clinical use. Fertil Steril.

[21] Viotti M, McCoy RC, Griffin DK, Spinella F, Greco E, Madjunkov M (2021). Let the data do the talking: the need to consider mosaicism during embryo selection. Fertil Steril.

[22] Leigh D, Cram DS, Rechitsky S, Handyside A, Wells D, Munne S (2022). PGDIS position statement on the transfer of mosaic embryos 2021. Reprod Biomed Online.

[23] Rienzi L, Cimadomo D, Delgado A, Minasi MG, Fabozzi G, Del Gallego R (2019). Time of morulation and trophectoderm quality are predictors of a live birth after euploid blastocyst transfer: a multicenter study. Fertil Steril.

[24] Harada Y, Maeda T, Fukunaga E, Shiba R, Okano S, Kinutani M (2020). Selection of high-quality and viable blastocysts based on timing of morula compaction and blastocyst formation. Reprod Med Biol.

[25] Mizobe Y, Ezono Y, Tokunaga M, Oya N, Iwakiri R, Yoshida N (2017). Selection of human blastocysts with a high implantation potential based on timely compaction. J Assist Reprod Genet.

[26] Ivec M, Kovacic B, Vlaisavljevic V (2011). Prediction of human blastocyst development from morulas with delayed and/or incomplete compaction. Fertil Steril.

[27] Iwata K, Yumoto K, Sugishima M, Mizoguchi C, Kai Y, Iba Y (2014). Analysis of compaction initiation in human embryos by using time-lapse cinematography. J Assist Reprod Genet.

[28] Ramos L, de Boer P (2011). The role of the oocyte in remodeling of the male chromatin and DNA repair: are events in the zygotic cell cycle of relevance to ART. Biennial Rev Infertility.

[29] Lagalla C, Tarozzi N, Sciajno R, Wells D, Di Santo M, Nadalini M (2017). Embryos with morphokinetic abnormalities may develop into euploid blastocysts. Reprod Biomed Online.

[30] Reignier A, Girard JM, Lammers J, Chtourou S, Lefebvre T, Barriere P (2019). Performance of Day 5 KIDScore morphokinetic prediction models of implantation and live birth after single blastocyst transfer. J Assist Reprod Genet.

[31] Petersen BM, Boel M, Montag M, Gardner DK (2016). Development of a generally applicable morphokinetic algorithm capable of predicting the implantation potential of embryos transferred on Day 3. Hum Reprod.

[32] Gazzo E, Pena F, Valdez F, Chung A, Bonomini C, Ascenzo M (2020). The Kidscore(TM) D5 algorithm as an additional tool to morphological assessment and PGT-A in embryo selection: a time-lapse study. JBRA Assist Reprod.

[33] Singla S, Iwamoto-Stohl LK, Zhu M, Zernicka-Goetz M (2020). Autophagy-mediated apoptosis eliminates aneuploid cells in a mouse model of chromosome mosaicism. Nat Commun.

[34] Bolton H, Graham SJL, Van der Aa N, Kumar P, Theunis K, Fernandez Gallardo E (2016). Mouse model of chromosome mosaicism reveals lineage-specific depletion of aneuploid cells and normal developmental potential. Nat Commun.

[35] Spinella F, Fiorentino F, Biricik A, Bono S, Ruberti A, Cotroneo E (2018). Extent of chromosomal mosaicism influences the clinical outcome of in vitro fertilization treatments. Fertil Steril.

[36] Munne S, Spinella F, Grifo J, Zhang J, Beltran MP, Fragouli E (2020). Clinical outcomes after the transfer of blastocysts characterized as mosaic by high resolution Next Generation Sequencing-further insights. Eur J Med Genet.

[37] Victor AR, Tyndall JC, Brake AJ, Lepkowsky LT, Murphy AE, Griffin DK (2019). One hundred mosaic embryos transferred prospectively in a single clinic: exploring when and why they result in healthy pregnancies. Fertil Steril.

[38] Munne S, Blazek J, Large M, Martinez-Ortiz PA, Nisson H, Liu E (2017). Detailed investigation into the cytogenetic constitution and pregnancy outcome of replacing mosaic blastocysts detected with the use of high-resolution next-generation sequencing. Fertil Steril.

[39] Zhang L, Wei D, Zhu Y, Gao Y, Yan J, Chen ZJ (2019). Rates of live birth after mosaic embryo transfer compared with euploid embryo transfer. J Assist Reprod Genet.

[40] Gardner DK, Lane M (1997). Culture and selection of viable blastocysts: a feasible proposition for human IVF?. Hum Reprod Update.

[41] Sui YY, Fu J, Zhang S, Li L, Sun XX (2022). Investigation of the role of X chromosome inactivation and androgen receptor CAG repeat polymorphisms in patients with recurrent pregnancy loss: a prospective case–control study. BMC Pregnancy Childbirth.

[42] Munne S, Wells D (2017). Detection of mosaicism at blastocyst stage with the use of high-resolution next-generation sequencing. Fertil Steril.

[43] Chuang TH, Chang YP, Lee MJ, Wang HL, Lai HH, Chen SU (2020). The Incidence of mosaicism for individual chromosome in human blastocysts is correlated with chromosome length. Front Genet.

