# Supplementary material for: The morphokinetic signature of human blastocysts with mosaicism and the clinical outcomes following transfer of embryos with low-level mosaicism
Source: J Ovarian Res. 2024 Jan 9;17:10. doi: 10.1186/s13048-023-01324-w (PMC10775590; doi:10.1186/s13048-023-01324-w)
Supplement: Supplementary file 1 — Additional file 1: Supplementary table 4. Clinical outcomes of the genotype of mosaicism after blastocyst transfers. [file 13048_2023_1324_MOESM1_ESM.docx]

| **Supplementary table 4. Clinical outcomes of the genotype of mosaicism after blastocyst transfers** | | | | |  |  |  |  |
| --- | --- | --- | --- | --- | --- | --- | --- | --- |
| No. | Genotype of Mosaicism | Mosaic type | Mosaicism rate | Female age at transfer (y) | Clinical outcomes | Gestational age at delivery  (week + day) | Birth weight (g) | Congenital anomaly |
| 1 | 30% mosaic gain of 9q12-q34.3 | segmental | 30% | 32 | Live birth | 39+4 | 3300 | None |
| 2 | 30% mosaic loss of chr4 | whole | 30% | 38 | Implantation fail |  |  |  |
| 3 | 20% mosaic loss of chr14 | whole | 20% | 37 | Implantation fail |  |  |  |
| 4 | 30% mosaic gain of chr1; 30% mosaic loss of chr7; 30% mosaic loss of chr9; 30% mosaic loss of chr18 | complex | 30% | 36 | Implantation fail |  |  |  |
| 5 | 30% mosaic loss of chr1 | whole | 30% | 27 | Clinical pregnancy fail |  |  |  |
| 6 | 30% mosaic loss of 8p23.3-q21.1 | segmental | 30% | 34 | Ectopic pregnancy |  |  |  |
| 7 | 50% mosaic loss of chr3 | whole | 50% | 38 | Implantation fail |  |  |  |
| 8 | 30% mosaic gain of 5q13.2-q35.3 | segmental | 30% | 31 | Clinical pregnancy fail |  |  |  |
| 9 | 30% mosaic gain of 13q21.31-q34 | segmental | 30% | 32 | Live birth | 38+4 | 3370 | None |
| 10 | 30% mosaic gain of chr5 | whole | 30% | 32 | Clinical pregnancy fail |  |  |  |
| 11 | 20% mosaic gain of chr1; 20% mosaic gain of chr2 | complex | 20% | 39 | Implantation fail |  |  |  |
| 12 | 30% mosaic loss of chr15 | whole | 30% | 34 | Implantation fail |  |  |  |
| 13 | 40% mosaic gain of chr19 | whole | 40% | 32 | Clinical pregnancy fail |  |  |  |
| 14 | 50% mosaic gain of 16p13.1 (~6.44Mb) | segmental | 50% | 31 | Implantation fail |  |  |  |
| 15 | 35% mosaic loss of 7q32.1-q36.3 | segmental | 35% | 31 | Clinical pregnancy fail |  |  |  |
| 16 | 25% mosaic gain of chr1 | whole | 25% | 28 | Biochemical pregnancy |  |  |  |
| 17 | 45% mosaic loss of 3q22.3-q29 | segmental | 45% | 39 | Live birth | 38+4 | 3200 | None |
| 18 | 30% mosaic loss of 7q22.3-q35 | segmental | 30% | 32 | Implantation fail |  |  |  |
| 19 | 30% mosaic gain of chr12 | whole | 30% | 34 | Live birth | 41 | 3000 | Hemivertebral deformity |
| 20 | 30% mosaic gain of chr3 | whole | 30% | 37 | Implantation fail |  |  |  |
| 21 | 30% mosaic loss of 8q11.21-q24.3 | segmental | 30% | 32 | Implantation fail |  |  |  |
| 22 | 30% mosaic loss of 5q33.2-q35.3 | segmental | 30% | 31 | Live birth | 37+4 | 2500 | None |
| 23 | 30% mosaic loss of chr18 | whole | 30% | 32 | Miscarriage |  |  |  |
| 24 | 50% mosaic loss of chr17 | whole | 50% | 33 | Live birth | 39 | 3450 | None |
| 25 | 30% mosaic loss of 9q13-q34.3 | segmental | 30% | 30 | Live birth | 38 | 3100 | None |
| 26 | 30% mosaic loss of chr8 | whole | 30% | 33 | Live birth | 38+2 | 2950 | None |
| 27 | 30% mosaic gain of chr4 | whole | 30% | 28 | Live birth | 39+2 | 3600 | None |
| 28 | 30% mosaic gain of 1p36.33-p12 | segmental | 30% | 36 | Live birth | 40+1 | 3800 | None |
| 29 | 30% mosaic loss of 4p16.3-p15.2 | segmental | 30% | 33 | Biochemical pregnancy |  |  |  |
| 30 | 40% mosaic gain of chr20 | whole | 40% | 40 | Implantation fail |  |  |  |
| 31 | 30% mosaic gain of 17q22-q24.2 | segmental | 30% | 40 | Implantation fail |  |  |  |
| 32 | 30% mosaic gain of chr19 | whole | 30% | 35 | Implantation fail |  |  |  |
| 33 | 40% mosaic loss of 12p13.33-p12.1 | segmental | 40% | 39 | Implantation fail |  |  |  |
| 34 | 30% mosaic loss of Xp22.33-p22.12 | segmental | 30% | 35 | Implantation fail |  |  |  |
| 35 | 30% mosaic loss of 5p15.33-p13.1 | segmental | 30% | 36 | Biochemical pregnancy |  |  |  |
| 36 | 30% mosaic gain of chr2 | whole | 30% | 32 | Live birth | 37 | 2900 | None |
| 37 | 30% mosaic gain of 7q11.1-q21.12 | segmental | 30% | 32 | Implantation fail |  |  |  |
| 38 | 30% mosaic loss of chr7 | whole | 30% | 42 | Implantation fail |  |  |  |
| 39 | 30% mosaic loss of chr22 | whole | 30% | 35 | Implantation fail |  |  |  |
| 40 | 35% mosaic loss of 7q33-q36.3 | segmental | 35% | 44 | Live birth | 38 | 3280 | None |
| 41 | 30% mosaic loss of chr5 | whole | 30% | 32 | Live birth | 39+3 | 3800 | None |
| 42 | 30% mosaic gain of 8q11.23-q24.3 | segmental | 30% | 29 | Live birth | 38+1 | 2500 | None |
| 43 | 30% mosaic loss of 7q31.1-q36.3 | segmental | 30% | 36 | Live birth | 40+2 | 3960 | None |
| 44 | 25% mosaic gain of chr10 | whole | 25% | 26 | Live birth | 37+5 | 3600 | None |
| 45 | 25% mosaic loss of chr13 | whole | 25% | 31 | Live birth | 38+5 | 2900 | None |
| 46 | 30% mosaic loss of chr7 | whole | 30% | 41 | Implantation fail |  |  |  |
| 47 | 30% mosaic loss of 9q33.2q34.3 | segmental | 30% | 40 | Live birth | 39+3 | 3700 | None |
| 48 | 30% mosaic loss of chr14 | whole | 30% | 35 | Live birth | 38+1 | 3120 | None |
| 49 | 40% mosaic loss of chr12 | whole | 40% | 36 | Implantation fail |  |  |  |
| 50 | 25% mosaic loss of chr7 | whole | 25% | 36 | Implantation fail |  |  |  |
| 51 | 30% mosaic loss of chr1; 30% mosaic loss of 17q23.3q25.3 | complex | 30% | 29 | Live birth | 39+2 | 2710 | None |
| 52 | 30% mosaic gain of chr4 | whole | 30% | 37 | Live birth | 38+2 | 3300 | None |
| 53 | 30% mosaic loss of 3q25.2q26.2 | segmental | 30% | 43 | Live birth | 38+3 | 3400 | None |
| 54 | 35% mosaic loss of chr9 | whole | 35% | 41 | Biochemical pregnancy |  |  |  |
| 55 | 30% mosaic gain of 2q11.2q37.3 | segmental | 30% | 32 | Live birth | 36 | 2320 | None |
| 56 | 40% mosaic loss of 8q21.2q24.3 | segmental | 40% | 34 | Implantation fail |  |  |  |
| 57 | 30% mosaic gain of 6q14.1q26 | segmental | 30% | 41 | Implantation fail |  |  |  |
| 58 | 30% mosaic gain of 5q11.2q12.2 | segmental | 30% | 42 | Implantation fail |  |  |  |
| 59 | 30% mosaic gain of 8q13.3q24.3 | segmental | 30% | 34 | Biochemical pregnancy |  |  |  |
| 60 | 30% mosaic gain of 11q13.2q14.1 | segmental | 30% | 30 | Implantation fail |  |  |  |
